# Firm bankruptcies and start-up activity in Switzerland during the COVID-19 crisis

**DOI:** 10.1186/s41937-022-00085-5

**Published:** 2022-03-09

**Authors:** Florian Eckert, Heiner Mikosch

**Affiliations:** KOF Swiss Economic Institute, ETH Zurich, Leonhardstrasse 21, Building LEE, 8092 Zurich, Switzerland

**Keywords:** Firm bankruptcies, Insolvencies, Excess mortality, Firm formations, Start-ups, Switzerland, COVID-19 crisis, Industry-level, Canton-level, E32, G33, M13

## Abstract

This paper examines the incidence of firm bankruptcies and start-ups in Switzerland based on unique register data. We propose to assess the frequency of bankruptcies over time using the concept of excess mortality. During the COVID-19 crisis in 2020 and the first half of 2021, bankruptcy rates were substantially lower as compared to the pre-crisis period. This holds across most industries and regions. The Great Recession and the Swiss Franc Shock showed reverse patterns. Bankruptcies dropped more in industries and cantons, in which the share of firms who received a COVID-19 loan is comparatively high. In winter 2021, bankruptcies rebounded strongly. Since summer 2020, the number of new firm formations has been significantly higher compared to the time before the crisis. This is also in contrast to the previous crises. The strong start-up activity is driven by industries where the pandemic induced structural adjustments.

## Introduction

The spread of the SARS-CoV-2 virus in early 2020 and the subsequent containment measures induced a dramatic collapse in economic activity, both in Switzerland and worldwide. Private households reduced their consumption activity due to lockdown restrictions, supply shortages and precautionary behavior. As a consequence, firms’ profits and demand expectations fell dramatically and business uncertainty increased (e.g., KOF, 2020). This led to public worries about a wave of firm bankruptcies in the near future. Media outlets and political players have been speculating whether or not a wave of bankruptcies will emerge.^1^ Against this background, we started in June 2020 to conduct a monthly monitoring of firm bankruptcies and start-up activity in Switzerland.^2^ In this paper, we describe our data and methods in detail and present an assessment of the bankruptcy and start-up dynamics during the COVID-19 crisis.

A major challenge for the near real-time assessment of business failures and start-ups is that the frequency of bankruptcies fluctuates strongly, even in normal times. Seasonal and cyclical patterns as well idiosyncrasies additionally complicate the near real-time evaluation. Hence, it is often hard to tell whether a strong increase at the current edge should be considered as worrisome or not. To overcome this challenge, we propose to apply the concept of excess mortality from the literature on human mortality, which received wide attention at the height of the COVID-19 pandemic (e.g., EuroMOMO, 2020). In accordance with this literature, we define exceptional excess mortality (undermortality) as a situation when the frequency of firm bankruptcies exceeds (falls below) the upper (lower) bound of a normality range around the trend. To our best knowledge, we are the first ones who propose to apply the excess mortality concept to the bankruptcy and start-up activity of the firm sector.^3^

A serious calculation of normality ranges is only possible based on a long data history. For this purpose, we collected the monthly number of firm bankruptcies and firm formations recorded in the Swiss Official Gazette of Commerce (Schweizerisches Handelsamtsblatt, Feuille officielle suisse du commerce, Foglio ufficiale svizzero di commercio) since the year 2000. We then constructed monthly time series on the frequency of bankruptcies and start-ups in the 8 Swiss greater regions, in the 26 Swiss cantons, and in the different industries of the Swiss economy. We also differentiate by age category and legal form. Using monthly data on the stock of firms, we further construct time series on bankruptcy and start-up rates. Since all legal bankruptcies and formations in Switzerland enter the Swiss Official Gazette of Commerce, our dataset covers basically the total population of bankruptcies and new formations. We are not aware of any other project internationally that monitors the frequency of bankruptcies and start-ups based on comparable data in terms of sample coverage, length of time series, and degree of disaggregation.

An extensive résumé of our empirical findings is provided in the conclusion of the paper. Here, we give a very brief summary: Bankruptcy rates in Switzerland were remarkably lower in 2020 and the first half of 2021 as compared to pre-crisis levels or long-term averages. This holds across all industry groups and greater regions of the Swiss economy. The finding stands in contrast to heightened (or at least not lowered) bankruptcy rates during and after previous crises. Three factors play a role for the different development during the current crisis. First, legal suspension measures delay at least some bankruptcies until today. Second, the COVID-19 loan programs gave firms at risk the opportunity to access fresh liquidity and prevent an insolvency. Bankruptcies dropped more strongly in industries where the share of firms with COVID-19 loans is high. The same association holds for a cross-canton comparison. Third, the broad use of the short-term work program resulted in a partial hibernation of the firm sector. The low bankruptcy rates reflect this hibernation. In winter 2021, bankruptcies increased strongly. It remains to be seen whether a prolonged period of increased insolvencies will follow. Furthermore, apart from an initial drop in April 2020 the number of new formations has been substantially higher during the years 2020 and 2021 than during the pre-crisis time. This is in sharp contrast to the subdued start-up activity during and after previous crises. Our findings suggest that the strong start-up activity is driven by industries who experience structural adjustments due to the pandemic. Notably, our findings on start-up activity are in line with recent evidence for the United States (Haltiwanger, 2021).

Our paper relates to a vivid international debate on the consequences of the COVID-19 crisis for bankruptcies and start-up activity (see, e.g., Gourinchas et al., 2020 for international evidence as well as Guerini et al., 2020 and Carletti et al., 2020 for evidence with French or Italian data, respectively). Some contributions recommend quick and bold interventions to prevent a surge in bankruptcies (e.g., Hanson et al., 2020; Demmou et al., 2020; Schivardi & Romano, 2020). Others advocate more cautious interventions (e.g., Bircan et al., 2020; Goodhart et al., 2020; Bailey et al., 2021). In view of the recently low levels of bankruptcies in many countries, the potential issue of “zombification” is fiercely discussed (e.g., Gobbi et al., 2020; Laeven et al., 2020; Cros et al., 2021).^4^ Another discussion concerns the adaption of bankruptcy laws and insolvency procedures given expectations of a resurgence of insolvencies (e.g., Greenwood et al., 2020; Demmou et al., 2021; Djankov & Zhang, 2021).^5^ In this paper, we are cautious regarding policy conclusions. Instead, our intention is to provide the reader with a detailed description of the bankruptcy and start-up dynamics in Switzerland during the COVID-19 crisis. We consider our monitoring as a necessary pre-condition, next to other data work, for well-informed policy recommendations.

Further, our paper relates to a (pre-COVID-19 crisis) literature on firm entries and exits over the business cycle (e.g., Caballero & Hammour, 1991, 2005; Caballero et al., 2008; Bilbiie et al., 2012; Varum & Rocha, 2012; Lee & Mukoyama, 2015; Daepp et al., 2015; Foster et al., 2016; Clementi & Palazzo, 2016). In line with this literature, we find that periods of economic expansion are usually associated with lower exit rates and higher entry rates than economic crisis periods. Our finding of a reversed pattern after February 2020 adds to the perception that the COVID-19 years 2020 and 2021 are a rather unusual period of time.^6^

The remainder of the paper is structured as follows. Section 2 describes the data on firm bankruptcies and firm formations used in this study. Section 3 discusses how we construct time-series based measures for excess mortality, undermortality, excess formation and underformation of firms. Section 4 provides a chronology of events and policy measures during the COVID-19 crisis. Section 5 presents the results. Section 6 summarizes the findings and provides conclusions.

## Data

**Fig. 1:**
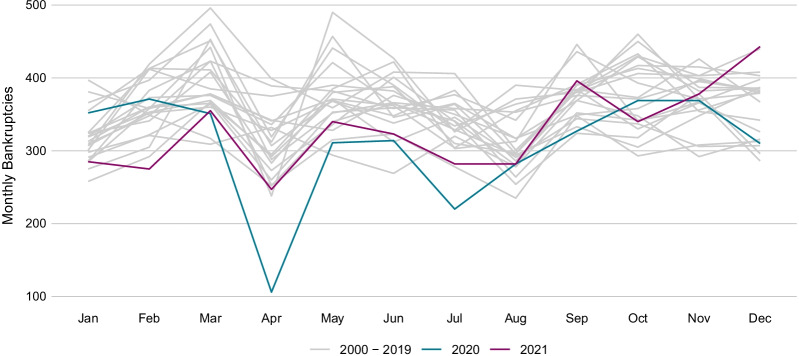
Seasonal pattern of firm bankruptcies in Switzerland. *Notes*: The figure shows the monthly seasonal pattern of total firm bankrupcties in Switzerland excluding SCO Art. 731b cases

The data for this study comprise the firm bankruptcies and formations recorded in the Swiss Official Gazette of Commerce (SOGC). Since all legal bankruptcies and formations in Switzerland enter the SOGC, the dataset covers basically the *total population* of bankruptcies and new formations. The data are provided by Dun & Bradstreet Schweiz AG, which collects the SOGC records at the firm level and pairs it with further firm-specific information . For this study, we use monthly time series on the frequency of firm bankruptcies and new firm formations in the 26 Swiss cantons and in the different industries of the Swiss economy since the year 2006 and in overall Switzerland since the year 2000. We further use monthly time series on the stock of firms in the different cantons and industries in order to calculate bankruptcy hazard rates. Figure 1 shows the seasonal pattern in bankruptcies, caused by, e.g., financial reporting periods and legal holidays.^7^

Regarding the bankruptcy data, three further points are noteworthy. First, in order to record a firm bankruptcy as early as possible, we count always the first announcement in the SOGC that indicates the bankruptcy of a firm. These announcements also include preliminary bankruptcy notices. The preliminary notices are sometimes revoked. If so, this usually happens within the next few months after publication of the notice. As a consequence, the bankruptcy time series are subject to slight data revisions (as is the case for many other macroeconomic time series, too). For this study, we use the final data vintages. Second, the bankruptcy of a company is sometimes published in the SOGC with a certain time delay. About half of the bankruptcies are published within 7 days of the opening of the bankruptcy proceedings, the vast majority within the first 4 weeks. Third, according to Article 731b of the Swiss Code of Obligations (SCO), the bankruptcy office can declare a firm as bankrupt due to organizational deficiencies. Such deficiencies might be a missing firm location or a government board, which is not constituted according to law.^8^ It is an open question whether bankruptcies due to organizational deficiencies should actually be counted as bankruptcies for the purpose of this study. On the one hand, the fact that a firm is declared as bankrupt due to organizational deficiencies does, prima facie, not mean that the firm is insolvent. On the other hand, grave organizational deficiencies could indicate that the firm is in bad economic health. Hence, an exclusion of these bankruptcy types risks to overestimate the health of the firm sector. To ensure a robust appraisal, we will in Sect. 5 present bankruptcy figures including and excluding liquidations due to organizational deficiencies.

Regarding the firm formation data, the following further points are worth mentioning. First, sole proprietorships need to be registered in the SOGC only if their annual turnover exceeds CHF 100,000. As a consequence, some sole proprietorships with small firms do not register at all. Alternatively, they register only substantial time after the actual firm establishment, for instance, when it turns out that the firm runs well. Second, a change in the legal form of a company is not counted as a new company formation. Third, in some countries and industries exists the practice to periodically liquidate and found firms anew in order to, e.g., get rid of outstanding debts. It is an open question to what degree this practice exists in Switzerland too.

## Modelling excess mortality of firms

In order to identify excess mortality, it is necessary to determine the expected number of bankruptcies in the absence of seasonal, cyclical and random fluctuations. We assume that each series $X_{t}$ can be decomposed multiplicatively according to $X_{t} = T_{t} C_{t} S_{t}E_{t}$, where $T_{t}$ is a trend component, $C_{t}$ is a cyclical component, $S_{t}$ is a seasonal component and $E_{t}$ is a remainder term, which we assume to be log-normally distributed (more on this assumption below). We take logarithms to account for larger variations in the components as the level of bankruptcies increases. Using lowercase letters to denote logs, i.e. $\log X_{t} = x_{t}$, each time series is then given by

1 $$\begin{aligned} x_{t}&= t_{t} + c_{t} + s_{t} + e_{t}, \quad e_{t} \sim {\mathcal {N}}(0,\sigma ^{2}), \end{aligned}$$

where $e_{t}$ is a normally distributed remainder term. We use a seasonal-trend decomposition based on locally estimated scatter plot smoothing (LOESS) (Cleveland et al., 1990) to extract the seasonal component $s_{t}$, the remainder $e_{t}$ and the local trend, given by $t_{t} +c_{t}$. We prefer STL-LOESS to X13-ARIMA-SEATS or related seasonality adjustment methods, since the latter are less stable when applied to volatile series with many zero values, occurring in some small aggregates. Next, we extract the long-run trend $t_{t}$ from the local trend, using the Hodrick–Prescott (HP) filter.^9^ For the smoothing parameter $\lambda$, Ravn and Uhlig (2002) suggest a value of 129,600 to capture fluctuations of monthly series at a business cycle frequency. This value, however, leads to residual cyclical patterns in the long-run trend, because business failures and formations occur at a lower frequency. We set $\lambda = 10^{6}$, which substantially reduces spurious cyclicality in the long-run trend.

The cyclical component $c_{t}$ can then be simply obtained by subtracting the long-run trend $t_{t}$ from the local trend. We refer to the difference between the seasonally adjusted number of bankruptcies and the long-run trend as the *excess mortality of firms*. Note that, according to this definition, excess mortality can also be negative, namely when the seasonally adjusted number of bankruptcies is lower than the trend. Negative excess mortality may also be called undermortality. Excess mortality can, equivalently, be described as the component of bankruptcies that is due to cyclical and random fluctuations.

2 $$\begin{aligned} x_{t} - t_{t} - s_{t} = c_{t} + e_{t}, \quad e_{t} \sim {\mathcal {N}}(0,\sigma ^{2}). \end{aligned}$$

We use $\sigma ^{2}$ to construct a probability range around the long-run trend. This interval describes the probabilities of firm bankruptcies and formations during normal times in the absence of cyclical fluctuations. The log-normality assumption for the remainder term $E_{t}$ may not always be plausible: for small aggregates with many zero observations, the bounds tend to become very narrow. Alternatively, we assume the remainder term $E_{t}$ to follow a Poisson distribution, where the rate parameter $\lambda$ varies over time and is equal to the respective trend value extracted from the HP Filter at each point in time. When the frequency series values are low (high), the ranges obtained from the Poisson distribution tend to be broader (narrower) than those obtained from the log-normal distribution. To ensure a conservative assessment of possible excess mortality, we always choose the wider one of the two ranges.^10^

The probability ranges may be interpreted as the “normal range” of firm bankruptcies/formations. If the frequency of bankruptcies in a period exceeds the upper bound of the range, we call this a situation of *exceptional excess mortality of firms*. For instance, if the number of bankruptcies is above the range in which the bankruptcy variable lies with a probability of 90%, there exists exceptional excess mortality beyond the 90% range. In contrast, a situation, where the frequency of bankruptcies falls below the lower bound of the range, is referred to as exceptional undermortality of firms. Notably, the probability ranges should not be interpreted as statistical confidence bands. Also, our criterion for excess mortality or undermortality is not a criterion for statistical significance, but a criterion for economic significance. In fact, the question of statistical significance is less relevant for us, since the studied time series include the total population of firm bankruptcies/formations in Switzerland (see Sect. 2).

The use of the terms excess mortality and undermortality is inspired by the literature on human excess mortality.^11^ However, there are differences as the latter literature deals with data on humans and conforms to the standards of empirical research in clinical biology, whereas we deal with firm data and conform to the standards of empirical macroeconomics. First, the human excess mortality literature controls for temperature and other factors affecting human mortality, whereas we seasonally adjust the data according to procedures used in economics. Second, the aforementioned literature employs different versions of the Poisson distribution in order to calculate normality ranges, while we opted for the mix of Poisson distribution and log-normal distribution described above. Third, the aforementioned literature defines the human excess mortality in any given period as the non-seasonally adjusted mortality (or the mortality controlled for specific factors) minus the expected number of deaths under normal conditions. This expected baseline is either determined using averages of past periods or is modeled using, e.g., trigonometric functions to express trends and seasonalities (Nielsen et al., 2018). Human excess mortality in clinical biology does, therefore, typically not account for a cyclical component with periodicity longer than 1 year. In contrast, we define the excess mortality of firms as the seasonally adjusted number of bankruptcies minus the trend, which results from adjusting the number of bankruptcies for cyclical patterns using the Hodrick–Prescott filter as described above.

## Chronology of events and policy measures

The first SARS-CoV-2 infection in Switzerland was recorded on 25 February 2020. In March, the virus spread quickly. Households responded by reducing their mobility and, as an immediate consequence, consumption activity dropped dramatically (Kraenzlin et al., 2020; Eckert & Mikosch, 2020). Restrictions on events and gatherings of persons were introduced in late February and early March, followed by a nationwide lockdown on 16 March. A decline in infections allowed authorities to reopen stores and schools on 29 April, followed by shops, restaurants, markets, museums and libraries on 11 May. Federal authorities quit the extraordinary situation in June, delegating responsibility to the cantons. Rules on hygiene and social distancing continued to remain in place. A second wave in the fall of 2020 prompted new containment measures: gatherings were restricted on 19 October, followed by capacity restrictions for stores and restaurants in early November. Restaurants and other public entertainment venues were closed in late December, followed by particular shops in mid-January 2021. Most restrictions were gradually eased during the spring of 2021.

**Fig. 2:**
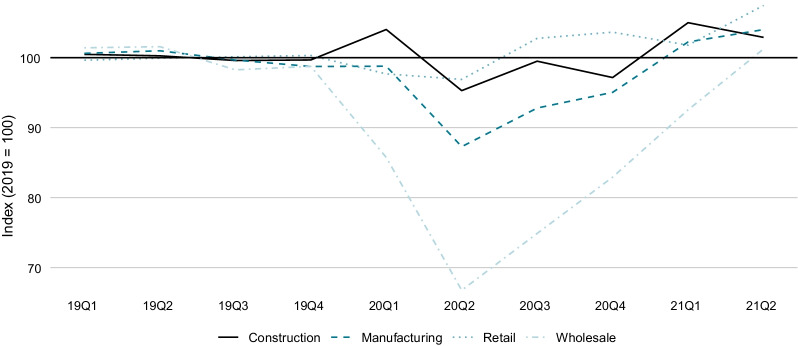
Quarterly revenues in selected industries. *Notes*: The figure shows the seasonally adjusted revenues in selected industries as compiled by the Swiss Federal Statistical Office in the quarterly Industry Production, Orders and Turnover Statistics (INDPAU). The data is indexed such that the average of 2019 is equal to 100

As a consequence of the containment measures and the drop in demand, many firms faced a collapse in revenues. Figure 2 shows the development of revenues in selected industries. Total turnover in the trade sector dropped by 12.6% in 2020Q1 and by 20.6% in 2020Q2 (all indicated numbers are quarter-on-quarter growth rates). The recovery back to the levels of the year 2019 took until 2021Q2. Further, total turnover in the manufacturing sector fell by 0.8% in 2020Q1 and by 11.0% in 2020Q2. It went back to the levels of 2019 in 2021Q1 only. Construction was hit to a lesser extent: revenues still grew by 4.4% in 2020Q1, fell by 8.4% in 2020Q2 and were back to the 2019 levels by the end of 2020.

Amid fears of a liquidity squeeze and a subsequent bankruptcy wave in the firm sector, the government authorities reacted quickly with legal and fiscal measures. Regarding legal measures, the Swiss Federal Council ordered a legal standstill for the period 19 March to 4 April 2020 and a subsequent debt collection holiday until 19 April. In addition, the obligation on companies to report over-indebtedness was suspended until 19 October. Further, the federal government took advantage of the parliament’s previous decision to expand the provisional debt restructuring moratorium option in case of a composition agreement from 4 to 8 months.^12^ Concretely, the government decided on 14 October to advance the enactment of this law change to 20 October. The rationale of these legal measures was to buy firms the time to adapt to the new situation without an immediate threat of having to declare bankruptcy, e.g., by adapting the business model or by reducing costs.

Regarding financial measures, on 20 March 2020 the Swiss Federal Council announced the launch of its COVID-19 loan program.^13^ Under this program, which ran from 26 March to 31 July 2020, companies were able to obtain loans secured in whole or in part by the Confederation from private banks at favorable conditions within a short period of time. The explicit purpose of the loans was to cover the companies’ running costs. Specifically, the federal state guaranteed 100% of the loan at an interest rate of currently 0% up to a maximum of CHF 500,000 or 10% of annual turnover (“Covid-19 credit”). Further, it provided a 85% loan guarantee at an interest rate of currently 0.5% per annum on the guaranteed portion of the loan from CHF 500,000 up to a maximum of CHF 20 million or 10% of annual turnover (“Covid-19 credit plus”). The term of the loans is 5 years or 7 years in cases of hardship. A total of 137’850 firms took out a loan with an average amount of CHF 122’910.^14^ In addition, from 7 May until 31 August 2020 the federal government ran a separate program to support bank loans for qualified start-ups.^15^ 359 loans were granted under this program. Furthermore, cantonal and federal authorities started in early 2021 to provide hardship aid for firms suffering from substantial decreases in revenue.

Another financial measure was a repeated expansion of the short-time working regime from March 2020 onwards. Specifically, the government introduced a summary settlement procedure to ease the administrative burden and speed up the payment process. In addition, it increased the maximum time period for access to short-time work compensation in several steps to currently 24 months. Further, the program was extended to persons with fixed-term contracts and to apprentices. The general purpose of these measures is to support affected companies with covering their fixed personnel costs, thereby, preventing losses and increases in unemployment.

Several cantons topped the aforementioned measures with their own initiatives. For instance, the canton of Zurich took a variety of measures such as the provision of additional loan guarantees for Zurich based companies and money provision for local municipalities to support self-employed persons. Also, the canton allowed companies to delay tax payments.^16^

An analysis on how these measures causally affected the bankruptcy dynamics during the COVID-19 crisis is beyond the scope of this paper. However, the descriptive evidence presented in the next section suggests that the measures were associated with a strong drop in bankruptcies in 2020 and 2021.

## Results

This section analyzes the developments of firm bankruptcies during the COVID-19 crisis at the sectoral, regional and aggregate level and puts them into historical perspective. The developments of new firm formations are discussed thereafter. The metrics evaluated in this section are calculated using the previously suggested excess mortality decomposition.

### Bankruptcies at the aggregate level

**Fig. 3:**
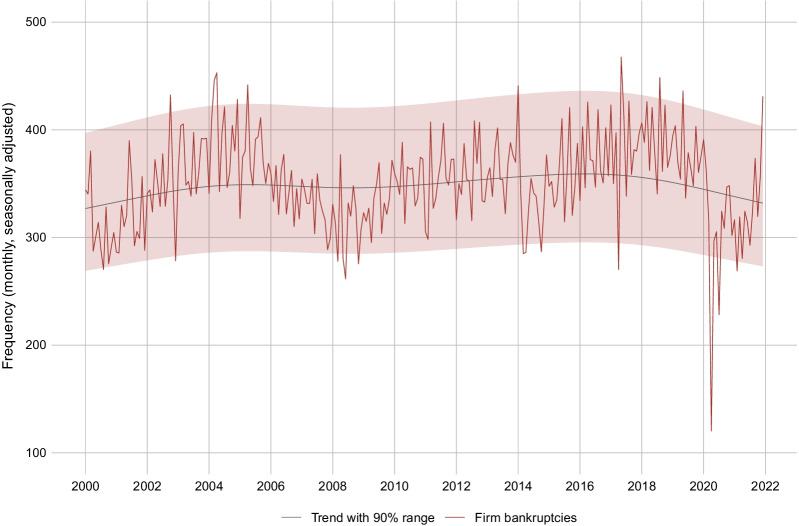
Monthly frequency of firm bankruptcies in Switzerland. *Notes*: The figure shows the monthly seasonally adjusted frequency of total firm bankrupcties in Switzerland excluding SCO Art. 731b cases, together with a trend and the 90% probability range

Figure 3 shows the monthly frequency of corporate bankruptcies in Switzerland since the year 2000. While the data is generally very volatile, extended periods of bankruptcies above or below trend are clearly visible. During 2002–2005, where economic growth was relatively weak in the aftermath of the Dot-com bubble, the frequency of bankruptcies tended to be above trend. After economic growth resumed in 2005, bankruptcies tended to be below trend until 2009. 2010–2014 was another above-trend phase, when the economy struggled following the Great Recession in 2008/09 and the European sovereign debt crisis in 2012. After bankruptcies had been at comparatively low levels in 2014 and 2015, they moved again above trend in 2016–2018. During this time period, a strong real exchange rate following the Swiss Franc Shock in 2015 put a burden on the economy. Notably, periods of exceptional excess mortality or undermortality beyond the 90% range are generally limited to 1 or 2 months only.

As can be seen from Fig. 3, the COVID-19 crisis came with a historically unprecedented slump in the number of bankruptcies in April 2020. The obvious reason for this slump are the legal standstill and the subsequent debt holidays, which put the bankruptcy activity into hibernation mode (see Sect. 4). In contrast to the general expectation, bankruptcies did not catch up after the legal freeze. Instead, they rebounded only partially and remained at rather low levels during the rest of 2020 and the first half of 2021. Only since summer 2021, bankruptcies tend back to pre-crisis levels with a strong increase at the current edge (December 2021).

**Fig. 4:**
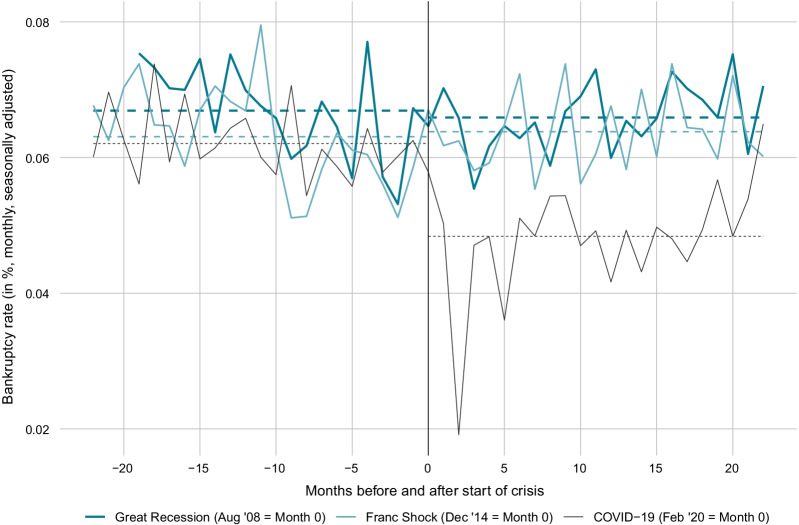
Comparison of bankruptcy rates before and after crisis periods. *Notes*: The figure shows the monthly bankruptcy rate (= monthly seasonally adjusted frequency of total firm bankrupcties excluding SCO Art. 731b cases divided by the total stock of firms) in Switzerland before and after the start of the Great Recession, the Swiss Franc Shock and the COVID-19 crisis. Month 0 is the last month before the crisis start. The dotted lines are the average bankruptcy rates over the 22 months before or after the respective crisis start

Figure 4 compares the monthly bankruptcy rates (number of bankruptcies divided by total stock of firms) before and after the start of the COVID-19 crisis with the bankruptcy rates before and after two preceding crises. The exact start dates (= month 1) of the crises are not always obvious. We chose those months as crisis starts, in which the estimated weekly Swiss GDP growth, according to Eckert et al. (2020), turned negative: August 2008 for the Great Recession, January 2015 for the Swiss Franc Shock, and March 2020 for the COVID-19 crisis. The dotted lines depict the bankruptcy rates on average over the 22 months before or after the respective crisis start. On average over the 22 months before the COVID-19 crisis, around 0.062% of all firms in Switzerland went bankrupt per month (0.75% annualized). This value is close to the long-term average.^17^ The bankruptcy rate dropped sharply after the start of the COVID-19 crisis. Only in the last month of 2021, it jumped to somewhat above pre-crisis levels. The monthly bankruptcy rate on average from March 2020 to December 2021 is 0.048% (0.58% annualized). Its difference to the pre-crisis mean bankruptcy rate is economically meaningful.^18^ Notably, this is also the case if one excludes the slumps in March and April 2020, caused by the legal freeze and the debt collection holidays. In comparison, bankruptcy rates increased on average after the Swiss Franc shock, albeit only marginally. Further, there was virtually no difference between the mean bankruptcy rates before and after the start of the Great Recession. The number of bankruptcies went down during the second half of 2008 and increased again afterwards.

Three factors potentially explain the weak bankruptcy activity during 2020 and the first half of 2021. First, it is well possible that the legal measures discussed in Sect. 4 delay bankruptcies until today. Specifically, the temporarily suspended obligation to report an over-indebtedness and the subsequent extension of the provisional debt restructuring moratorium option from 4 to 8 months in case of a composition agreement gave over-indebted firms the opportunity to prevent a bankruptcy declaration during 2020 and the first half of 2021. Second, the federal government’s COVID-19 loan program, which ran from March to July 2020, gave firms easy access to long-term loans on favorable terms (see Sect. 4). It can be presumed that especially firms at risk (due to the COVID-19 shock or other reasons) furnished fresh liquidity.^19^ This reduces the risk of a temporary spike in bankruptcies and will smooth bankruptcies over time.^20^ Indeed, there is some evidence that, on the cantonal level as well as on the industry level, a high share of firms with COVID-19 loans is associated with a comparatively strong drop in bankruptcy rates after the start of the crisis (see below). Third, the short-term work program, which got expanded and simplified as compared to previous crises, allowed affected firms to cover most of their labor costs. This might have resulted in a hibernation of parts of the firm sector: on the one hand, revenues went down due to the economic crisis and, on the other hand, costs went down as well due to the short-term work program. The low bankruptcy numbers might be considered as a reflection of this presumed hibernation.^21^

The series in Figs. 3 and 4 exclude bankruptcies according to SCO Art. 731b (see Sect. 2). For robustness, we also consider the series including these types of bankruptcies (see Fig. 15 in the Appendix). The series including Art. 731b cases suggests the same conclusions than the series excluding Art. 731b cases discussed above. In particular, there is no evidence that bankruptcies during the COVID-19 pandemic occurred via Art. 731b cases.

It is possible that firms voluntarily exited the market due to the COVID-19 crisis rather than filing for bankruptcy. Consequently, the firm closures would not show up in the bankruptcy registers. Hong and Saito (2021) find this voluntary exit effect for Japan. To see whether the effect is also present in Switzerland, we study the deletions from the SOGC over time. On average over the past 10 years, around 23% of the deletions from the SOGC resulted from bankruptcy cases. The share typically fluctuates between 15 and 30%. We find no evidence that the number of deletions of firms from the SOGC was particularly high (or low) since the beginning of the COVID-19 pandemic (see Fig. 16 in the Appendix).

As will be discussed in the next section, the drop in bankruptcies is especially strong in Construction and Crafts. To check whether our aggregate findings are driven by this sector, we iterate Fig. 4 excluding the sector. It turns out that the exclusion of Construction and Crafts makes hardly any difference (see Fig. 17 in the Appendix).

### Disaggregate evidence on bankruptcies

While there is currently no evidence for a bankruptcy wave at the aggregate level, the COVID-19 crisis has hit Swiss firms quite differently, and, hence important disparities might exist across industries, regions and firm types. This section presents evidence on firm bankruptcies at the industry and regional level as well as for different age categories and legal types.

#### Sectoral evidence

**Fig. 5:**
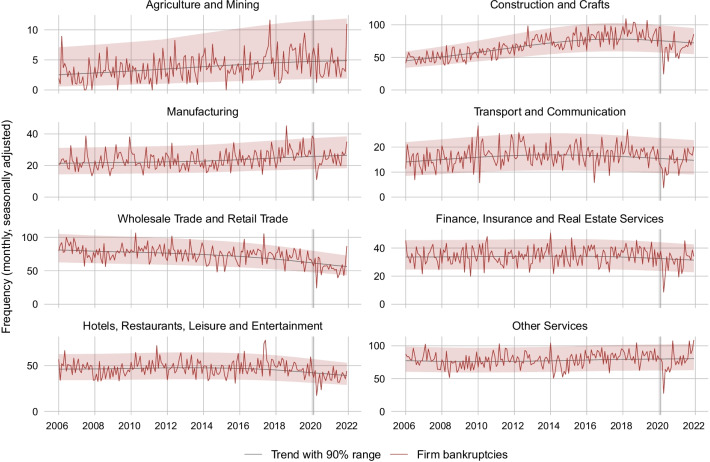
Monthly frequency of firm bankruptcies at the industry level. *Notes*: The figure shows the monthly seasonally adjusted frequency of firm bankrupcties in different sectors of the Swiss economy excluding SCO Art. 731b cases, together with a trend and the 90% probability range. Industries are grouped together according to economic similarity. The vertical line marks the last month before the start of the COVID-19 crisis (February 2020)

Figure 5 shows the number of corporate bankruptcies over time in various industry groups. The strong drop in bankruptcies in April 2020, following the legal freeze and the debt collection suspension, is visible throughout all industries. While bankruptcies rebounded quickly back to trend in most sectors, they stayed below trend for several months in Transport and Communication, Manufacturing as well as in Other Services. Since the start of the COVID-19 crisis, the following industries have experienced exceptional excess mortality beyond the 90% range: Other Services in October 2020, September 2021 and December 2021, Finance, Insurance and Real Estate Services in June 2021, and Wholesale and Retail Trade in November and December 2021.

**Fig. 6:**
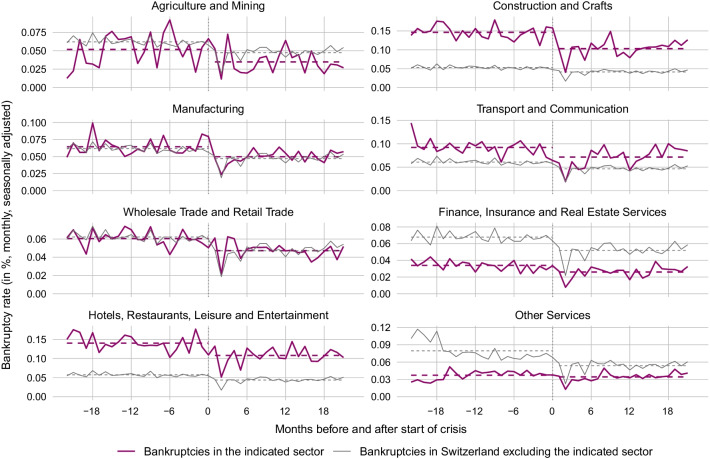
Bankruptcies in different industries before and after start of COVID-19. *Notes*: The figure shows the monthly bankruptcy rate (= monthly seasonally adjusted frequency of firm bankrupcties excluding SCO Art. 731b cases divided by the stock of firms) in different industries of the Swiss economy before and after the start of the COVID-19 crisis as compared to the bankruptcy rate in the respective rest of the economy. The last month before the crisis start (= month 0) is February 2020. The dotted lines are the average bankruptcy rates over the 22 months before or after the crisis start

**Fig. 7:**
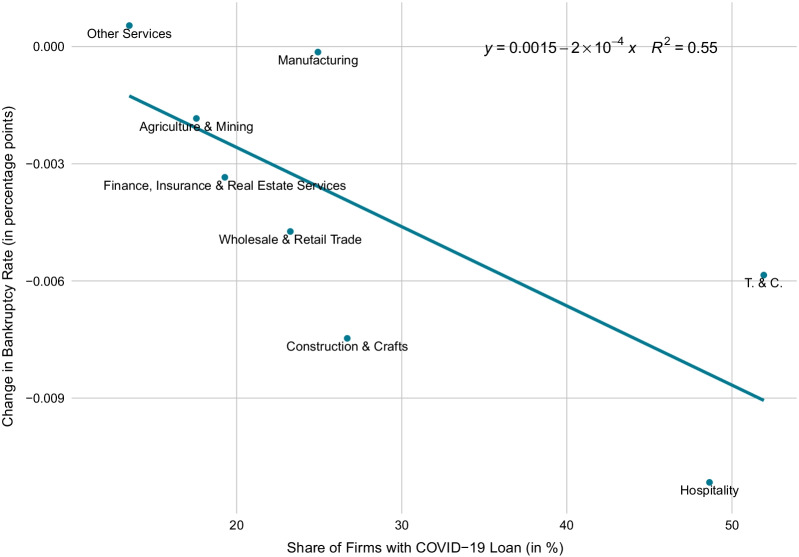
Relationship of COVID-19 loans and bankruptcies across sectors. *Notes*: The scatter plot shows the correlation between the share of firms who took out COVID-19 loans and the change in bankruptcy rates across sectors. The change in bankruptcy rates is given by the difference in average bankruptcy rates 22 months before and after the start of the COVID-19 crisis. The results are robust to choosing a 12-month or 18-month comparison window. T. & C. means transport and communication. Hospitality includes hotels, restaurants, leisure and entertainment

Figure 6 compares the bankruptcy rate in each industry during the months before and after the start of the COVID-19 crisis (red lines) with the bankruptcy rate in the respective rest of the Swiss economy before and after the crisis start (grey lines). The dashed lines depict the 22-month average bankruptcy rate before the start of the crisis (May 2018 to February 2020) and from the crisis start onward (March 2021 to December 2021). Surprisingly, as can be seen from a comparison of the red dashed lines before and after the crisis start, the average bankruptcy rate fell across all industry groups. Nevertheless, a comparison between industry and rest-of-economy changes in average bankruptcy rates reveals some discrepancies. For Construction and Crafts, the average bankruptcy rate fell substantially more than in the rest of the economy. The difference amounts to 0.031 percentage points (pp).^22^ This is also the case for Transport and Communication (0.003 pp), Agriculture and Mining (0.002 pp) as well as for Hotels, Restaurants, Leisure and Entertainment (0.018 pp), despite the fact that economic activity in the latter industry group was severely harmed by the sanitary restrictions. The cross-industry comparison in Fig. 7 suggests that a high share of firms with COVID-19 loans is associated with a comparatively strong drop in bankruptcy rates after the start of the crisis.

The decrease in bankruptcies for Manufacturing are pretty much equal to the change in the respective rest of the economy. The industry groups, in which bankruptcy rates dropped less than in the rest of the economy, are: Wholesale and Retail Trade (− 0.003 pp), Finance, Insurance and Real Estate Services (− 0.008 pp), and Other Services (− 0.021 pp). These industries were likely to benefit less from direct fiscal support measures, because many firms in these industries were not affected directly by the legal shutdown. Still, revenue in these industries dropped strongly as consumers voluntarily reduced their consumption activity in the face of the pandemic. Note that all these finding do not change when including SCO Art. 731b cases into the bankruptcy figures.

#### Bankruptcies by age category and legal form

**Fig. 8:**
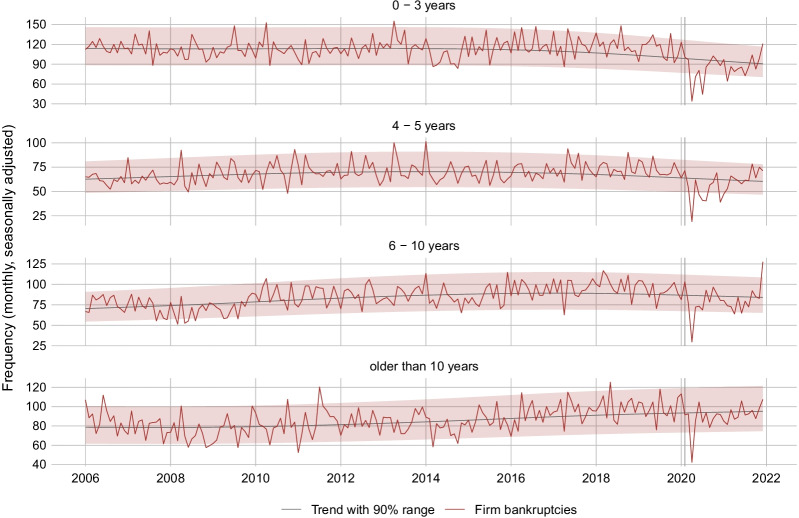
Firm bankruptcies by age category. *Notes*: The figure shows the monthly seasonally adjusted frequency of total firm bankrupcties in Switzerland excluding SCO Art. 731b cases dissagragated by age category

In the policy discussion on firm bankruptcies, it has sometimes been conjectured that the COVID-19 crisis threatens small or young firms more than bigger or older firms (e.g., Finanz und Wirtschaft, 2021). The reasoning behind this argument is that small and young firms often have less financial reserves than bigger and more established firms. While we have no solid information on the size (in terms of turnover or number of employees) of the bankrupt firms, we do so for their age. It turns out that, in contrast to our prior expectation, the decrease in firm bankruptcies during the COVID-19 crisis was especially driven by younger firms (see Fig. 8). Specifically, monthly bankruptcies of firms with age 0–3 years or 4–5 years fell by 27.0% or 21.2%, respectively, on average during March 2020 to December 2021 as compared to May 2018 to February 2020. Monthly bankruptcies of 6–10 year old firms fell by 16.1%, and bankruptcies of firms older than 10 years decreased by 9.4% only. The picture is not altered when changing the comparison window to, e.g., 12 or 18 months. The explanation of this finding is not clear. One reason might be that young firms are often also small firms. These firms participated more in the COVID-19 loan program due to a lack of alternative funding sources and the favorable funding conditions. In contrast, larger loans were only partially guaranteed and were subject to checks by the issuing bank (see Sect. 4).^23^

We also differentiate by the legal form of the bankrupt firms. Over the past 10 years, 46% of all bankrupt firms were limited liability companies, 26% were sole proprietorships and another 26% were public limited companies. All other legal forms, e.g., private limited partnership companies, co-operatives, public institutions, foundations and clubs, made up only 2% of all bankrupt firms. Monthly bankruptcies of sole proprietorships fell by 31.3% on average during March 2020 to December 2021 as compared to May 2019 to February 2020. Bankruptcies of limited liability companies and public limited companies decreased by 12.7% and 17.5% (see also Fig. 19 in the Appendix). Altering the comparison window does not change the difference between sole proprietorships and the other two categories.

#### Regional evidence

**Fig. 9:**
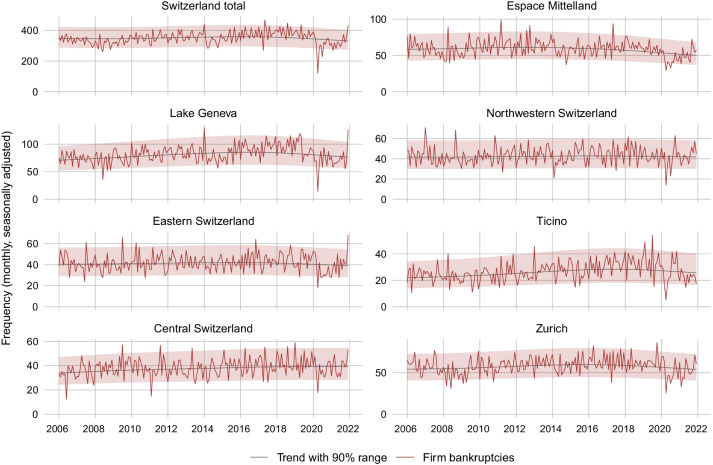
Monthly frequency of firm bankruptcies at the regional level. *Notes*: The figure shows the monthly seasonally adjusted frequency of firm bankrupcties in the Swiss greater regions excluding SCO Art. 731b cases, together with a trend and the 90% probability range

Figure 9 presents the frequency of corporate bankruptcies in the Swiss greater regions over time.^24^ The slump in April 2020 occurred in all greater regions. In the Lake Geneva region, Northwestern Switzerland, and Central Switzerland, bankruptcies rebounded quickly back to trend during the summer of 2020. In contrast, in the Espace Mittelland, Eastern Switzerland, Ticino and Zurich, the normalization occurred in fall or even winter. Until fall 2021, the only episodes of exceptional excess mortality beyond the 90% range are Northwestern Switzerland in October 2020 and Espace Mittelland in August 2021. In Winter 2021, all regions except the Ticino experienced a sharp increase in bankruptcies. Figure 18 in the Appendix contains the bankruptcy series at the cantonal level. None of the cantons has seen a wave of bankruptcies so far. However, bankruptcies increased significantly in several cantons in winter 2021 (Aargau, Appenzell Ausserrhoden, Bern, Genève, Schwyz, St. Gallen, Thurgau, Vaud, Zug, Zurich).

**Fig. 10:**
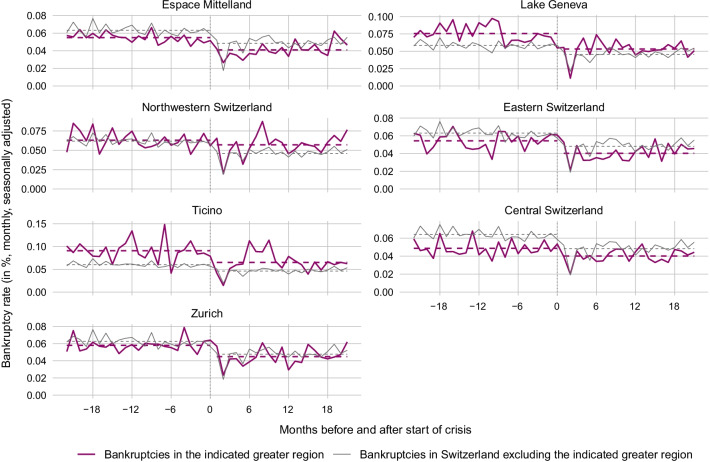
Regional bankruptcy rates before and after start of COVID-19. *Notes*: The figure shows the monthly bankruptcy rate (= monthly seasonally adjusted frequency of firm bankruptcies excluding SCO Art. 731b cases divided by the stock of firms) in the Swiss greater regions before and after the start of the COVID-19 crisis as compared to the bankruptcy rate in the respective rest of the economy. The last month before the crisis start (= month 0) is February 2020. The dotted lines are the average bankruptcy rates over the 22 months before or after the crisis start

Figure 10 compares the regional bankruptcy rates before and after the start of the COVID-19 crisis (red lines) with the bankruptcy rate in the respective rest of the Swiss economy before and after the crisis start (grey lines). Again, the dashed lines depict the 22-month average bankruptcy rate before the start of the crisis (May 2018 to February 2020) and from the crisis start onward (March 2020 to December 2021). Notably, as revealed by a comparison of the red dashed lines before and after the crisis start, the average bankruptcy rate fell in all greater regions.

**Fig. 11:**
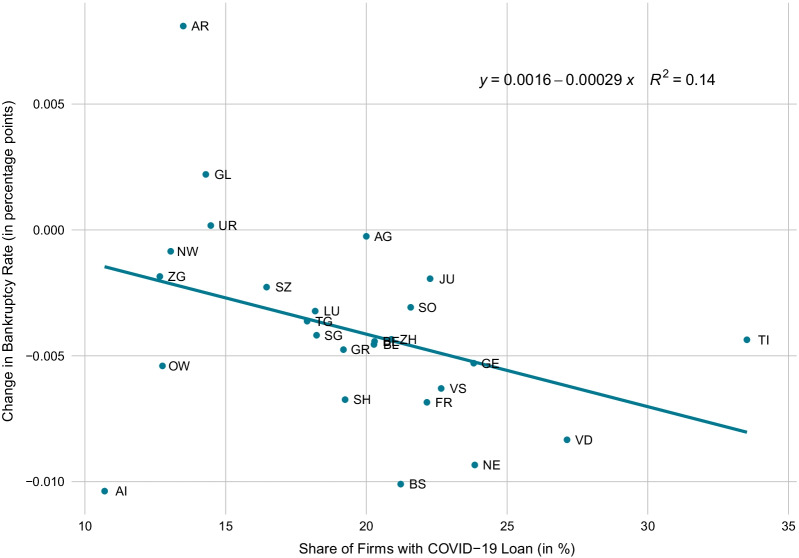
Relationship of COVID-19 loans and bankruptcies across cantons. *Notes*: The scatter plot shows the correlation between the share of firms who took out COVID-19 loans and the change in bankruptcy rates across cantons. The change in bankruptcy rates is given by the difference in average bankruptcy rates 22 months before and after the start of the COVID-19 crisis. The results are robust to choosing a 12-month or 18-month comparison window. Abbreviations: Aargau (AG), Appenzell Ausserrhoden (AR), Appenzell Innerrhoden (AI), Basel-Landschaft (BL), Basel-Stadt (BS), Bern (BE), Fribourg (FR), Genève (GE), Glarus (GL), Graubünden (GR), Jura (JU), Luzern (LU), Neuchâtel (NE), Nidwalden (NW), Obwalden (OW), Schaffhausen (SH), Schwyz (SZ), Solothurn (SO), St. Gallen (SG), Thurgau (TG), Ticino (TI), Uri (UR), Valais (VS), Vaud (VD), Zug (ZG) and Zürich (ZH)

In order to detect regional discrepancies, we compare the regional pre-crisis-to-crisis differences to the respective rest-of-economy pre-crisis-to-crisis difference. For instance, in Ticino the average bankruptcy rate dropped comparatively strongly despite the fact that this region was hit hard by the first COVID-19 wave. In contrast, in Northwestern Switzerland the drop was comparatively weak, although these regions were hit less hard at least during the first wave. One possible explanation is as follows: Firms in regions, which were hit comparatively strongly by the pandemic, took out COVID-19 loans more readily (see Fuhrer et al., 2020). This induced a general drop in bankruptcies in these regions. Indeed, according to the cross-canton comparison in Fig. 11, a high share of firms with COVID-19 loans in a canton is associated with a comparatively strong drop in bankruptcy rates after the start of the crisis.

For robustness, we repeat the analysis including SCO Art. 731b cases. The findings are quite similar and the general picture, according to which the COVID-19 crisis is associated with a drop in bankruptcies throughout all Swiss regions, does not change.

### Start-up activity

**Fig. 12:**
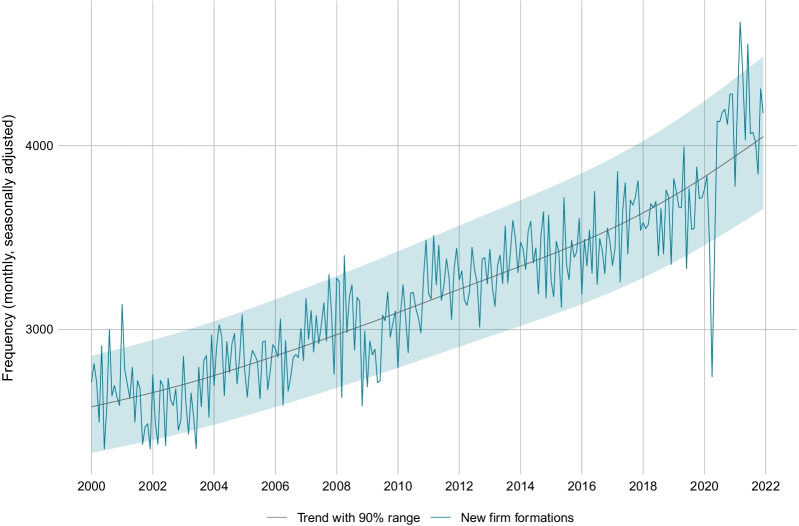
Monthly frequency of firm start-ups in Switzerland. *Notes*: The figure shows the monthly seasonally adjusted frequency of new firm formations in Switzerland, together with a trend and the 90% probability range

The number of firms leaving specific markets is an important indicator for structural changes. Similarly informative is the number of firms entering a market, which is usually a signal for improved business opportunities due to cyclical upturns or structural changes in a specific industry. In this section, we look at the start-up activity during the COVID-19 crisis as defined by new registrations in the SOGC. Figure 12 shows a drop in new formations in April 2020, most likely due to the legal freeze at the start of the pandemic. While this initial decline resembles the trajectory of bankruptcies, new formations recovered quickly and remained close to the upper end of the normal range during most of 2020. The number of firms entering the market was even above the normal range in early 2021.

**Fig. 13:**
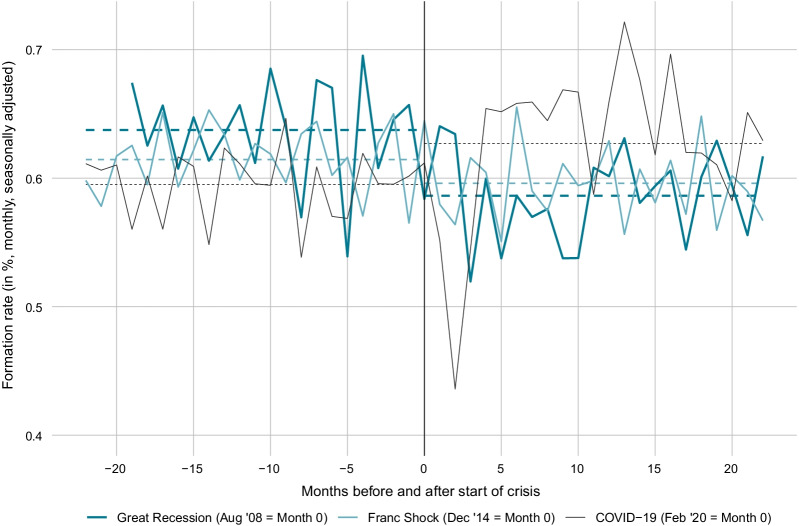
Comparison of start-up activity before and after crisis periods. *Notes*: The figure shows the monthly start-up rate (= monthly seasonally adjusted frequency of total firm start-ups divided by the total stock of firms) in Switzerland before and after the start of the Great Recession, the Swiss Franc Shock and the COVID-19 crisis. Month 0 is the last month before the crisis start. The dotted lines are the average bankruptcy rates over the 22 months before or after the respective crisis start

**Fig. 15:**
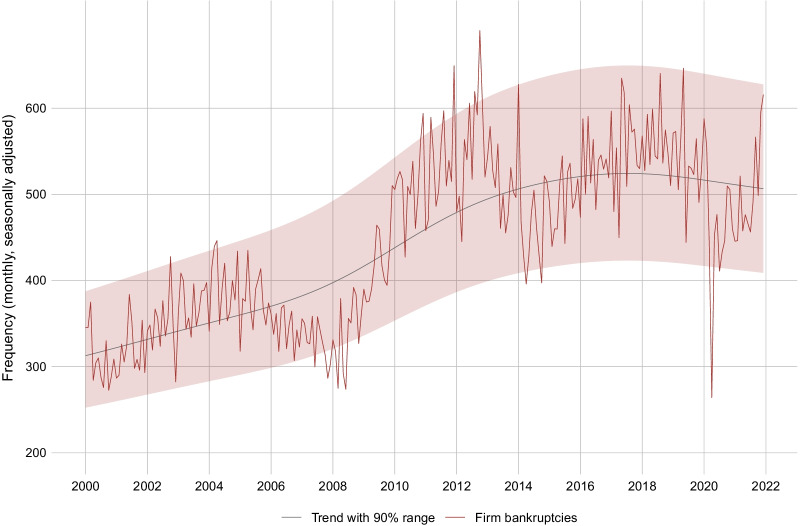
Firm bankruptcies in Switzerland Including SCO Art. 731b Cases. *Notes*: The figure shows the monthly seasonally adjusted frequency of total firm bankrupcties in Switzerland including SCO Art. 731b cases, together with a trend and the 90% probability range. Note that most of the trend increase from 2008 to 2012 was due to a law change which stipulated the counting of SCO Art. 731b cases as bankruptcies

**Fig. 16:**
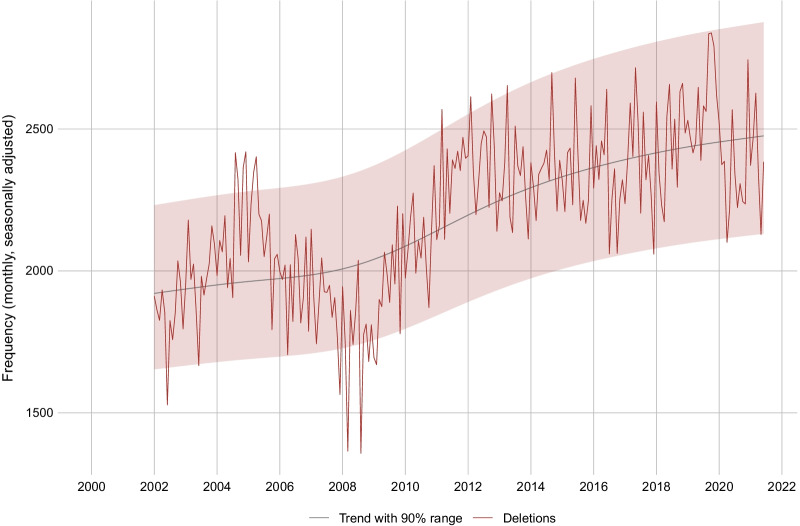
Deletions of firms from the Swiss Official Gazette of Commerce. *Notes*: The figure shows the monthly seasonally adjusted frequency of deletions of firms from the Swiss Official Gazette of Commerce, together with a trend and the 90% probability range. Note that most of the trend increase from 2008 to 2012 was due to a law change which stipulated the counting of SCO Art. 731b cases as bankruptcies and the deletion of Art. 731b cases from the Official Gazette of Commerce

**Fig. 17:**
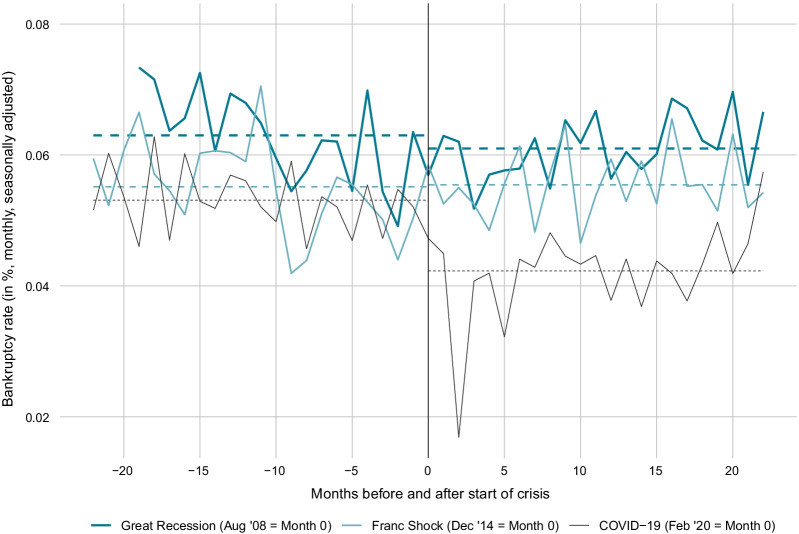
Bankruptcy rate comparison excluding construction and crafts. *Notes*: The figure shows the monthly bankruptcy rate in the Swiss economy excluding firms in Construction and Crafts before and after the start of the Great Recession, the Swiss Franc Shock and the COVID-19 crisis. Month 0 is the last month before the crisis start. The dotted lines are the average bankruptcy rates over the 22 months before or after the respective crisis start

**Fig. 18:**
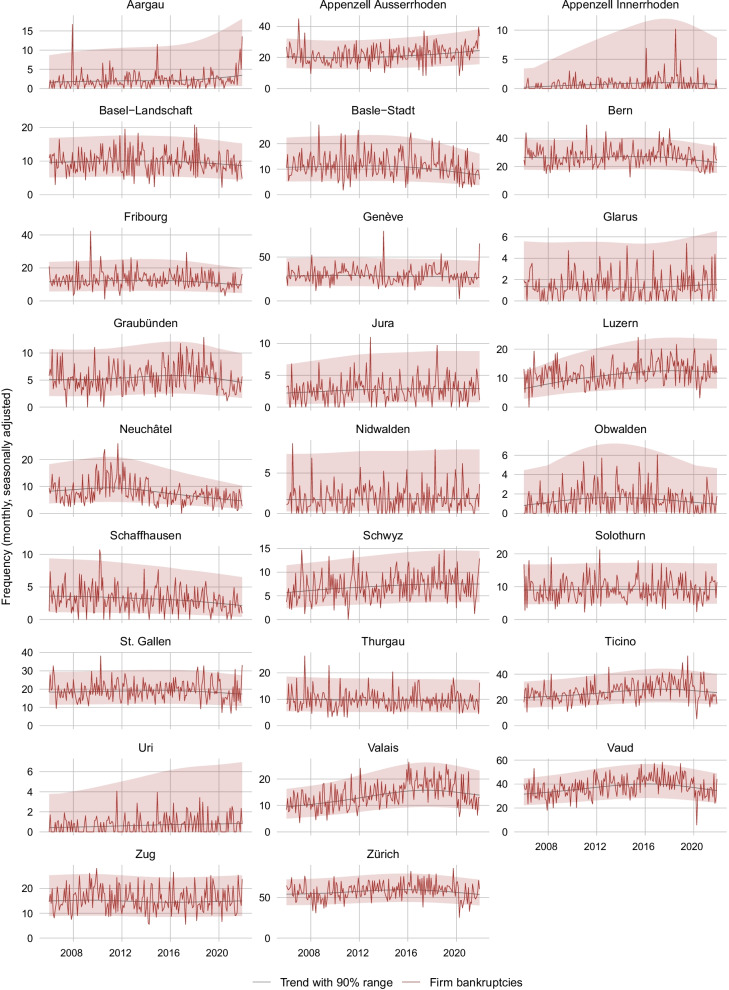
Monthly frequency of firm bankruptcies at the cantonal level. *Notes*: The figure shows the monthly seasonally adjusted frequency of firm bankrupcties in the Swiss cantons excluding SCO Art. 731b cases, together with a trend and the 90% probability range

**Fig. 19:**
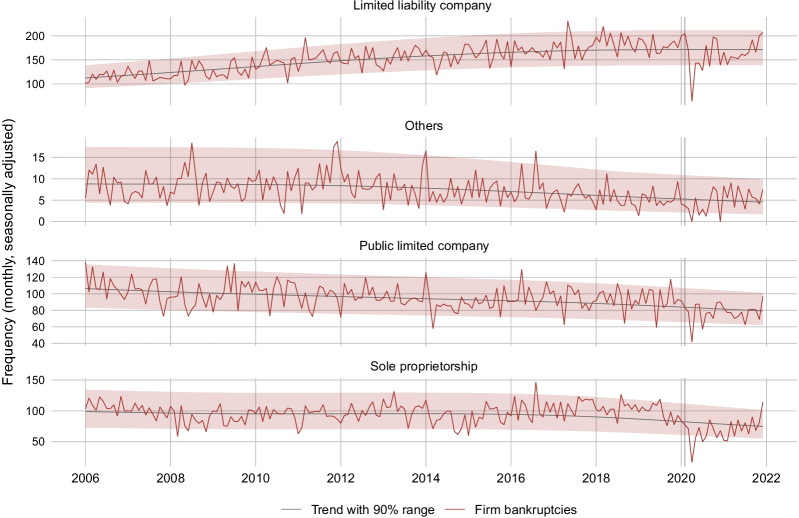
Firm bankruptcies by legal form. *Notes*: The figure shows the monthly seasonally adjusted frequency of total firm bankrupcties in Switzerland excluding SCO Art. 731b cases dissagragated by the legal form of the firms

The thriving start-up activity in the second half of 2020 and the first half of 2021 stands in contrast to the current cyclical position of the Swiss economy and in contrast to the experience from previous crises. As revealed by Fig. 13, the formation rate was subdued following the Great Recession as well as following the Swiss Franc Shock. In contrast, the formation rate during the COVID-19 crisis was substantially higher as compared to the pre-crisis time.

**Fig. 20:**
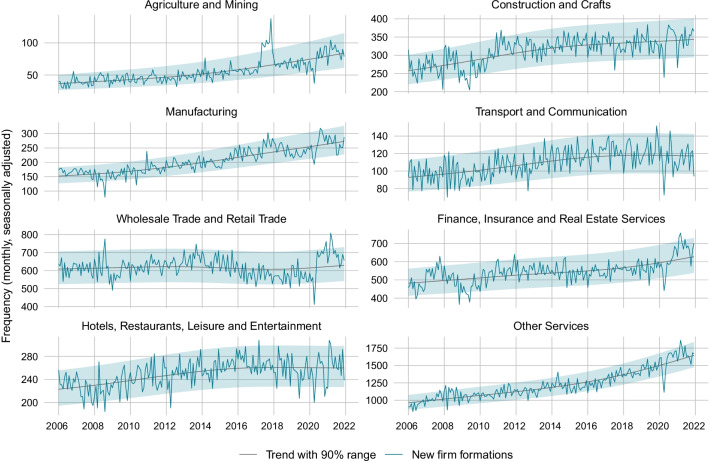
Monthly frequency of firm start-ups at the sectoral level. *Notes*: The figure shows the monthly seasonally adjusted frequency of new firm formations in the sectors of the Swiss economy excluding SCO Art. 731b cases, together with a trend and the 90% probability range. Sectors are grouped together according to economic similarity

**Fig. 21:**
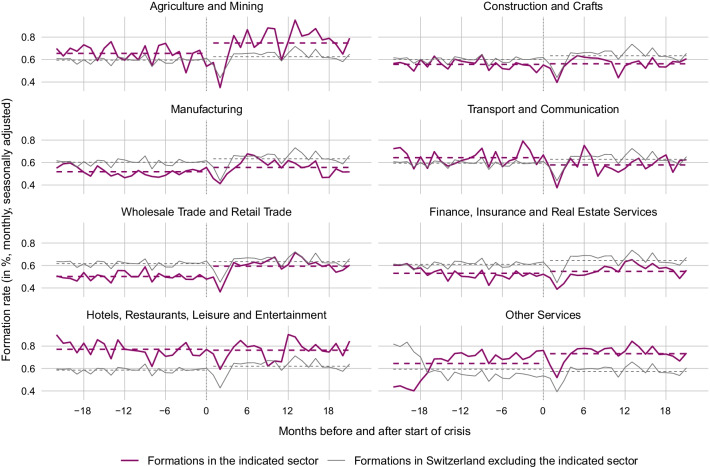
Start-up rates in different sectors before and after start of COVID-19. *Notes*: The figure shows the monthly start-up rate (= monthly seasonally adjusted frequency of new firm formations divided by the stock of firms) in the sectors of the Swiss economy before and after the start of the COVID-19 crisis as compared to the start-up rate in the respective rest of the economy. The last month before the crisis start (= month 0) is February 2020. The dotted lines are the average bankruptcy rates over the 22 months before or after the crisis start

**Fig. 22:**
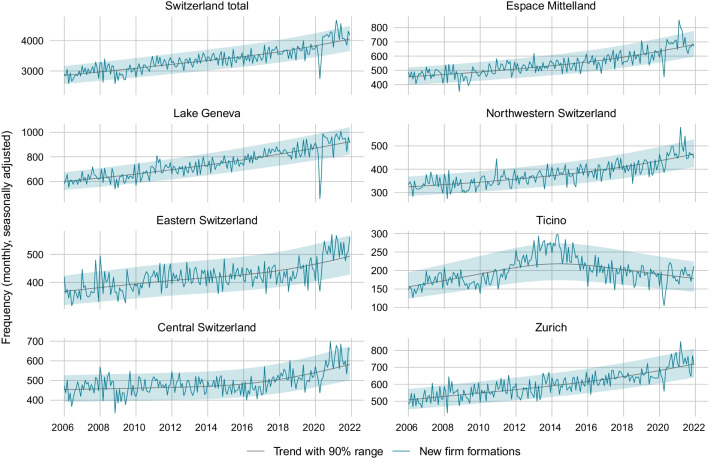
Monthly frequency of firm start-ups at the regional level. *Notes*: The figure shows the monthly seasonally adjusted frequency of new firm formations in the Swiss greater regions excluding SCO Art. 731b cases, together with a trend and the 90% probability range

**Fig. 23:**
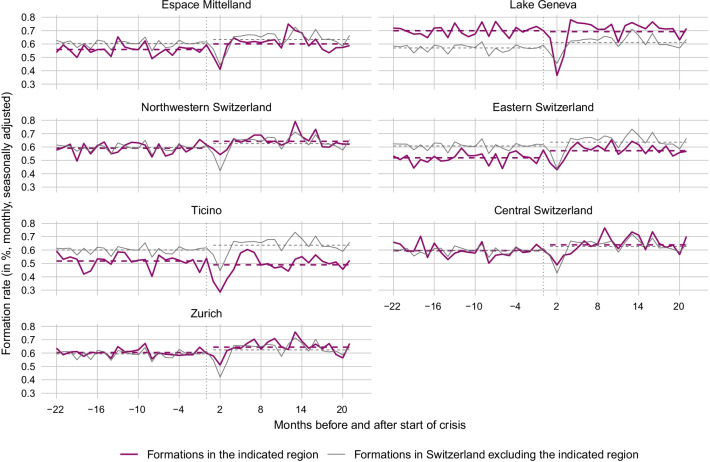
Regional start-up rates before and after start of COVID-19. *Notes*: The figure shows the monthly start-up rate (= monthly seasonally adjusted frequency of new firm formations divided by the stock of firms) in the Swiss greater regions before and after the start of the COVID-19 crisis as compared to the start-up rate in the respective rest of the economy. The last month before the crisis start (= month 0) is February 2020. The dotted lines are the average bankruptcy rates over the 22 months before or after the crisis start

Importantly, as only firms, who already existed on 1 March 2020, had access to the COVID-19 loan program, the surge in new firm formations cannot be attributed to this liquidity measure. A disaggregate analysis reveals that many increases in new formations occur in industries that are likely to undergo lasting structural adjustments as a result of the pandemic. This is visible, for instance, in wholesale and retail trade, where the shift to e-commerce has promoted the creation of new firms. Figures 20, 21, 22, 23 in the Appendix provide results for industry aggregates and regional aggregates.

## Conclusion

In this paper, we analyzed the frequency of firm bankruptcies and start-ups in Switzerland in order to monitor the economic impact of the COVID-19 pandemic. We did this on a disaggregate level (industries, cantons and greater regions) and also differentiated by age category and legal form. The data were extracted at monthly frequency from the Swiss Official Gazette of Commerce and cover the total population of firm bankruptcies and new firm formations. The collected monthly time series start in the year 2000 or 2006. Based on the long data history, we constructed probability ranges around a trend to determine time periods of exceptional excess mortality or undermortality of firms. This is helpful for a near real-time assessment in view of strong fluctuations in the time series.

We find that the legal freeze ordered by the Federal Council in March 2020 and the subsequent suspension of debt collection in April 2020 were followed by a historically unprecedented slump in the total number of bankruptcies as well as in the bankruptcy rate. In summer 2020, the frequency of bankruptcies partially rebounded but stayed substantially below pre-crisis levels. From March 2020 to December 2021, 0.58% of all firms in Switzerland went bankrupt on average per annum. In contrast, the average per annum bankruptcy rate over the 22 months before the crisis was 0.75%. The development stands in sharp contrast to the Swiss Franc Shock and the Great Recession, where bankruptcies increased or remained basically unchanged after the crisis start. Only at the current edge (December 2021) the bankruptcy rate jumped somewhat above the pre-crisis level (which is close to the long-term average).

We further find that the average bankruptcy rate fell across all industry groups during the crisis phase as compared to the pre-crisis phase. There is some tentative evidence that bankruptcies fell comparatively less in industries, who were less affected by the legal lockdown measures. One explanation for this counter-intuitive finding might be as follows: Because many firms in these industries were not legally obliged to close during the lockdown in spring 2020, they benefited less from direct fiscal support. Still, they encountered substantial revenue losses as households voluntarily reduced their mobility and consumption activity in the face of the pandemic. Regarding the regional data, we find that, on average from March 2020 to December 2021, all Swiss greater regions experienced a drop in their bankruptcy rate relative to the respective 22-month pre-crisis average. The drop was comparatively strong in Ticino and comparatively weak in Northwestern Switzerland. This finding is despite the fact that Ticino was affected earlier and stronger by the pandemic than the latter two regions. Another finding is that young firms experienced a stronger drop in bankruptcies during the crisis than old firms. While monthly bankruptcies of firms with age 0–5 years fell by around 27% during March 2020 to December 2021 as compared to the 22 months before the crisis, the bankruptcies of firms older than 10 years fell by around 9% only. This finding might be attributed to the COVID-19 loan program, which was especially targeted to small firms (with size being correlated with age).

Three factors have likely contributed to the low bankruptcy rates during 2020 and most of 2021. (1) Legal delay: The temporarily suspended obligation to report an over-indebtedness and the subsequent extension of the provisional debt restructuring moratorium option have possibly delayed bankruptcies until recently. (2) Loan glut: The government’s COVID-19 loan program gave firms easy access to cheap long-term loans. We find preliminary evidence that, on the cantonal level as well as on the industry level, a high share of firms, who took out COVID-19 loans, is associated with a comparatively strong drop in bankruptcy rates during the crisis. (3) Hibernation: As the crisis started and revenues dropped, many firms resorted to the short-term work program to cover their labor costs. This induced a partial hibernation of the firm sector, which is reflected in the low bankruptcy numbers. Each of the aforementioned factors has different implications for the future bankruptcy dynamics: The more relevant is the legal delay factor, the more likely will be a catch-up bankruptcy wave in the near future. In contrast, the more important is the loan factor, the less likely will be a speedy resurgence of bankruptcies with, however, risks for the future. Further, the more relevant the hibernation factor is, the more likely it is that the bankruptcy numbers return soon to normal levels.^25^

As regards the start-up activity in Switzerland during the COVID-19 crisis, we find that the number of newly registered firms dropped in April 2020 but then recovered quickly and remained very high throughout 2020 and 2021. The average formation rate during March 2020 to December 2021 was substantially higher than during the 22 months prior to the crisis. This stands in contrast to the Great Recession and the Swiss Franc Shock, where new firm formations were clearly subdued during and after the crisis phase. Our finding reveals once again that the COVID-19 crisis is quite special in historical comparison. It is important to note that only firms, who were already established on 1 March 2020, could participate in the COVID-19 loan program. Hence, it is unlikely that the surge in new firm formations is due to the increase in loan supply. Rather, our analysis suggests that new formations occured especially in industries who undergo structural adjustments as a result of the pandemic.

## Figures and Tables

**Fig. 14:**
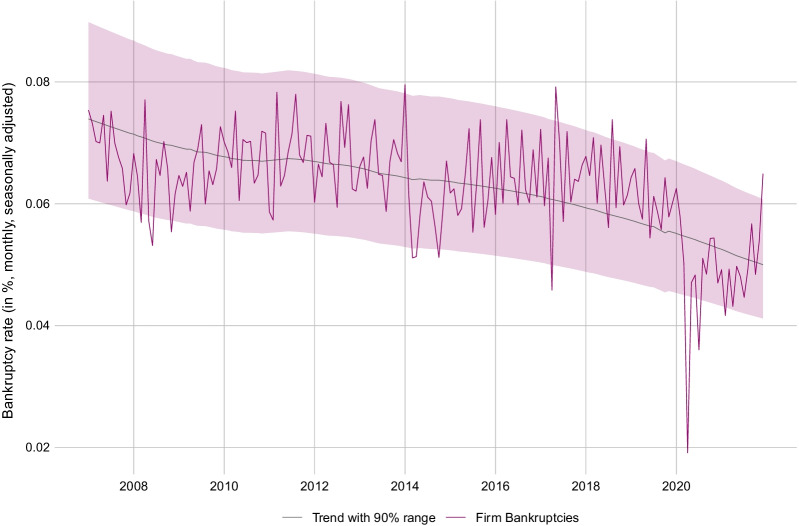
Monthly firm bankruptcy rate in Switzerland. *Notes*: The figure shows the monthly bankruptcy rate (= monthly seasonally adjusted frequency of total firm bankrupcties excluding SCO Art. 731b cases divided by the total stock of firms) in Switzerland over time. The stock of firms are available since September 2007 only, which limits the history of the time series. The time series seems to have a downward trend, however, this might be driven by cyclical movements both at the beginning of the series (Great Recession) and at the current end of the series (COVID-19 crisis)

## Data Availability

The data used for this paper are openly available from the Swiss Official Gazette of Commerce.

## Appendix

The appendix contains additional figures that have been referenced in the main text (see Figs. 14, 15, 16, 17, 18, 19, 20, 21, 22, 23).

## Abbreviations

APA: Administrative procedure act
HP Filter: Hodrick–Prescott filter
INDPAU: Industry production, orders and turnover statistics
LOESS: Locally estimated scatter plot smoothing
SCO: Swiss code of obligations
SOGC: Swiss official gazette of commerce

## Footnotes

1 See NZZ am Sonntag, 12 April 2020, Tagesanzeiger, 28 May 2020, Le Bilan, 10 June 2020 and many more. According to Heinz Karrer, the president of the Swiss corporate union Economiesuisse, in an interview with the Tagesanzeiger on May 29, 2020 “a giant wave of bankruptcies is currently rolling towards Switzerland”.

2 We disseminate the monitoring results via press statements, newsletter and bulletin articles of the KOF Swiss Economic Institute, ETH Zurich (e.g, Eckert et al., 2021). For the period July to December 2020, we recorded around 150 newspaper citations (0.83 citations per day). Also radio stations have been reporting on the monitoring (e.g., RTS Radio La 1ère, 2021a, 2021b).

3 Notably, while the aforementioned definition generally conforms with the literature on human excess mortality, the actual conceptualization of excess mortality in this paper is not exactly the same (see Sect. 3).

4 Interestingly, Hong and Saito (2021) report that, while bankruptcy rates in Japan have been extremely low during the crisis, a “cleansing effect” occurs via voluntarily firm exit. In contrast, we do not find evidence that firm deletions from the Swiss Official Gazette of Commerce were particularly high (or low) since the beginning of the COVID-19 pandemic.

5 Müller and Ammann (2021) analyze in detail the demography of the firm sector in Switzerland. They also address the effect of the COVID-19 crisis in 2020. Related to the aforementioned debate on bankruptcy reform, the authors advocate efficiency improvements in the Swiss institutional framework for the establishment and the closing of firms through, e.g., more ambitious digitalization.

6 Using Swiss data, Buehler et al. (2012) study the geographic determinants of firm bankruptcies. They find, amongst others, that bankruptcy rates are lower in regions with favorable business conditions and in central municipalities of agglomerations.

7 According to Art. 22a Administrative Procedure Act (APA), statutory or official periods do not run for two weeks around Easter, from 15 July to 15 August and from 18 December to 2 January.

8 Specifically, the article states: “If the company lacks one of the prescribed bodies or if one of these bodies is not lawfully constituted, a shareholder, a creditor or the Registrar of Companies may request the judge to take the necessary measures. The judge may in particular: ...dissolve the company and order its liquidation in accordance with the provisions on bankruptcy”.

9 The long-run trend of a series heavily depends on which trend extraction method is actually applied. We chose the HP Filter, because it still seems to be a widely used standard in macroeconomics. See, however, Hamilton (2018) for a critique of the HP Filter. For robustness checks, we also experimented with other filter and moving average methods.

10 In any case, both with the log-normal distribution and the Poisson distribution, the bounds of the probability range cannot be negative. This makes these two distributions popular for the work with count data such as the number of bankruptcies or start-ups.

11 See, e.g., Checchi and Roberts (2005), Nielsen et al. (2018, 2019).

12 See Art. 293a Swiss Debt Enforcement and Bankruptcy Law (Bundesgesetz über Schuldbetreibung und Konkurs/Loi fédérale sur la poursuite pour dettes et la faillite/Legge federale sulla esecuzione e sul fallimento). The parliament’s decision was part of a stock corporation law revision, which was launched independently of the COVID-19 crisis.

13 See https://covid19.easygov.swiss/en.

14 This number is calculated from the entire loan volume, not just the guaranteed share. 136’717 firms participated in the program Covid-19 credit with an average loan volume of CHF 101’919. 1’133 firms participated in the program Covid-19 credit plus with an average loan volume of CHF 2’655’919. For reasons of convenience, in the rest of the paper we address both programs jointly by using the term COVID-19 loan program.

15 See https://covid19.easygov.swiss/en/startup-guarantees.

16 See https://www.zh.ch/de/gesundheit/coronavirus/unternehmen-und-selbstaendige/unterstuetzung-und-finanzhilfen.html.

17 Figure 14 in the Appendix shows the long-run history of the monthly bankruptcy rate in Switzerland. On average since the beginning of the series in 2007, 0.064% of all firms in Switzerland went bankrupt per month (0.74% per year). The rate usually varies between 0.08% and somewhat below 0.06%.

18 Note that since the bankruptcy rate time series includes the total population of firm bankruptcies, all differences in the mean values must be considered as statistically significant, but they are not always economically meaningful.

19 Brlhart et al. (2020) analyze the response of self-employed workers to the COVID-19 crisis. They find, amongst others, that previously indebted self-employed took out COVID-19 loans more readily than those who were free of debt before the start of the crisis. Fuhrer et al. (2020) employ a comprehensive firm-level data set and study determinants for the participation in the COVID-19 loan program. They find several determining factors: the exposure to lockdown restrictions, the regional affection by the pandemic, the liquidity situation and firm age (being a proxy for financial vulnerability). In contrast, they find no clear evidence that firm indebtedness played a role for participation in the program.

20 Notably, the smoothing over time can be considered as a desirable policy effect of the program.

21 The question of the causal relevance of the aforementioned factors calls for further research. This is, however, beyond the scope of the present paper. Exploiting cross-cantonal variation, Kaufmann (2020) studies the causal effect of the loan supply shock implied by the COVID-19 loan program. He finds that the loan program substantially mitigated the increase in unemployment during the crisis.

22 The bankruptcy rate in Construction and Crafts is generally much higher than in the other sectors (except for the hospitality sector, i.e. Hotels, Restaurants, Leisure and Entertainment). Possible explanations are that there exist many small, liquidity-constrained firms in this sector and that the sector is characterized by a high competitive pressure and a risky environment. The observed drop in bankruptcies might thus be due to the COVID-19 loan program, which provided the liquidity-constrained firms with a liquidity injection and, as a consequence, generally lowered bankruptcies resulting from temporary illiquidity. Another hypothesis is that many individuals in this sector repeatedly file for bankruptcy frivolously and then restart their business to get rid of their debts. These frivolous bankruptcies might have been put on hold after the start of the pandemic to benefit from furlough and other compensation payments and from COVID-19 loans.

23 Our results relate to Dörr et al. (2020) who find that the fiscal rescue policy in Germany during the COVID-19 crisis led to a “backlog of insolvencies” that is particularly pronounced among financially weak, small firms.

24 The Swiss greater regions are composed by cantons as follows: Northwestern Switzerland: Aargau, Basel-Landschaft, Basel-Stadt; Espace Mittelland: Bern, Fribourg, Jura, Neuchâtel, Solothurn; Lake Geneva: Geneva, Valais, Vaud; Zurich: Zurich; Ticino: Ticino.

25 A causal analysis of the actual relevance of the three factors and possibly other explanations goes beyond the scope of this paper and calls for new research.
